# Ketamine for treatment-resistant obsessive-compulsive disorder: Double-blind active-controlled crossover study

**DOI:** 10.1177/02698811241301215

**Published:** 2024-11-28

**Authors:** Ben Beaglehole, Paul Glue, Shona Neehoff, Shabah Shadli, Neil McNaughton, Bridget Kimber, Chrissie Muirhead, Aroha de Bie, Rachel Day-Brown, Natalie J Hughes-Medlicott

**Affiliations:** 1 Department of Psychological Medicine, University of Otago, Christchurch, New Zealand; 2 Department of Psychological Medicine, University of Otago, Dunedin, New Zealand; 3 Department of Psychology, University of Otago, Dunedin, New Zealand; 4 School of Psychology, Charles Sturt University, Bathurst, NSW, Australia; 5 School of Pharmacy, University of Otago, Dunedin, New Zealand

**Keywords:** Ketamine, obsessive-compulsive disorder

## Abstract

**Background:**

Obsessive-Compulsive Disorder (OCD) may respond to ketamine treatment.

**Aim:**

To examine the responsiveness and tolerability of treatment-refractory OCD to intramuscular (IM) ketamine compared to IM fentanyl.

**Methods:**

This was a randomised double-blind psychoactive-controlled study with single doses of racemic ketamine 0.5 mg/kg, 1.0 mg/kg or fentanyl 50 µg (psychoactive control). Pre-dosing with 4 mg oral ondansetron provided nausea prophylaxis. Eligible participants were aged between 18 and 50 years with severe treatment-resistant OCD. The primary efficacy measure was the Yale-Brown Obsessive-Compulsive Scale (Y-BOCS). Tolerability was measured with the Clinician-Administered Dissociative States Scale (CADSS). Repeated measures analysis of variance with orthogonal polynomial trends was used to assess the effect of drug treatment on Y-BOCS and CADSS scores.

**Results:**

Twelve participants were randomised and 10 completed the study (7 females, 3 males, mean age 33 years). Two participants dropped out due to not tolerating dissociative effects associated with the study medication. The reductions in Y-BOCS scores were greater and statistically dose-related for both ketamine doses than fentanyl (dose [linear], *F*(1, 9) = 6.5, *p* = 0.031). Score changes for all treatments were maximal at 1–2 h with a steady separation of scores out to 168 h. Ketamine was associated with short-term dissociative and cardiovascular effects.

**Conclusions:**

We provide further preliminary evidence for the efficacy and tolerability of IM ketamine in an outpatient cohort of OCD. Additional work is required to establish the optimal dosing regimen and longer-term role of ketamine for OCD. These findings are encouraging given the well-known limitations that exist for treatments in this area.

## Introduction

Obsessive-Compulsive Disorder (OCD) is a mental disorder characterised by intrusive, unwanted and persistent thoughts (obsessions) and/or repetitive behaviours that the person feels driven to perform to reduce anxiety or distress (compulsions) (American Psychiatric Association, 2013). Reported lifetime prevalence rates for OCD vary considerably but are typically in the 0.5%–3% range (Fontenelle et al., 2006). OCD is burdensome: it causes greater interference with life than other anxiety disorders (Wells et al., 2006). Quality of life in OCD patients is substantially impaired; to a similar level as major depression and schizophrenia (Macy et al., 2013). Additionally, OCD is frequently co-morbid with depression, other anxiety disorders and neurodevelopmental disorders (Sharma et al., 2021).

Conventional treatments for OCD include antidepressant medications and psychotherapy, often in combination (Hirschtritt et al., 2017). Despite adequate medication trials, many patients still report residual impairing symptoms (Hirschtritt et al., 2017). Response rates to behavioural therapy, cognitive therapy or cognitive behavioural therapy are reported to be higher than with medications although clinical trials typically included participants who were already taking stable doses of antidepressants (Skapinakis et al., 2016). Medications and therapy both require many weeks to establish efficacy; and approximately one-quarter of OCD patients do not respond (Hirschtritt et al., 2017). Given the limitations of existing treatments and the extent of treatment-resistant OCD, new treatment options are desirable.

Ketamine is an N-methyl-D-aspartate (NMDA) receptor antagonist with many additional actions, and strong evidence for rapidly acting short-term treatment of treatment-refractory Major Depressive Disorder and increasing evidence in other disorders (Walsh et al., 2021). We are aware of one previous randomised controlled trial (RCT) evaluating ketamine for OCD. This evaluated 15 participants with OCD and compared a single intravenous (IV) infusion of 0.5 mg/kg ketamine against placebo infusion (Rodriguez et al., 2013). The infusions were spaced at least a week apart. There was a 50% response rate in the group treated with ketamine compared to 0% responders in the placebo group. Significant carryover effects were present meaning data from the second phase of the crossover could not be analysed. Rodriguez et al. (2015) reported a nearly identical sample supplemented by two additional participants but was focussed on reporting neurochemical as opposed to clinical effects of ketamine (Rodriguez et al., 2015). Rodriguez et al. (2017) attempted to evaluate the benefits of intranasal ketamine for OCD but had difficulty recruiting patients for intranasal ketamine treatment. Only one participant received intranasal ketamine and they did not respond to the treatment.

Further work evaluating ketamine for OCD is desirable. Key issues are the robustness and specificity of the ketamine response for OCD, optimal dosing and duration of treatment. Here, we report an RCT comparing two ketamine doses with fentanyl control in a community OCD sample.

## Materials and methods

The protocol and consent forms for this study were approved by the Central Health and Disability Ethics Committee (19/CEN/21). The study was registered with the Australian and New Zealand Clinical Trial Registry (ACTRN12619000311156). The wider study included the recruitment of patients with treatment-resistant major depressive disorder, treatment-resistant post-traumatic stress disorder, OCD and spider phobia in separate cohorts, to evaluate the effects of ketamine on EEG biomarkers (right frontal theta power) (Shadli et al., 2022); only changes in OCD symptom ratings, safety and tolerability from the OCD cohort are reported in this paper. Given varying definitions of treatment resistance in the context of anxiety disorders (Bokma et al., 2019), we operationalised this to be failure to respond to adequate trials of at least two prior conventional pharmacological and one psychological treatment.

This was a randomised double-blind psychoactive-controlled study in patients with treatment-resistant DSM-5 OCD. The study was undertaken in two community settings (Dunedin and Christchurch, New Zealand). The CONSORT checklist (Schulz et al., 2010) details the location of key design features (Supplemental Data File: CONSORT checklist). Participants were interviewed using a structured clinical interview (Sheehan et al., 1998). Inclusion criteria included having a Yale-Brown Obsessive-Compulsive Scale (Y-BOCS) (Goodman et al., 1989) score of >26, aged between 18 and 50 years, having good overall health and having had an unsatisfactory response to at least two prior antidepressant treatments and at least one relevant psychotherapy. Included participants were required to not have a Montgomery Asberg Depression Rating Scale (Montgomery and Asberg, 1979) score of >20 at screening. Other exclusion criteria included evidence of severe or chronic medical disorders, past or current diagnoses of schizophrenia, bipolar disorder or current psychotic symptoms, current significant suicidal ideation, patients who were pregnant or lactating, patients with substance use disorder or dependence in the last 6 months, and prior history of seizures or head injury. Ethnicity was ascertained by self-report and from health records. Participants provided signed informed consent before screening and were assessed as suitable to participate based on a review of medical history, safety laboratory tests (complete blood count, electrolytes, pregnancy test for patients who were capable of becoming pregnant), negative urine drug screening and vital signs. Participants were asked to provide a referral from a GP or psychiatrist who knew them well and could confirm the medical diagnosis and prior treatment. Participants were permitted to remain on current medication regimens and to continue with ongoing psychotherapy; however, no new treatments were to be started or changed during the study.

Study treatments were single doses of racemic ketamine 0.5 mg/kg, 1.0 mg/kg or fentanyl 50 µg (psychoactive control). These were administered as intramuscular (IM) injections in the deltoid muscle. Study drugs were given by P.G. or B.B. according to a computer-generated random code with balanced randomisation, using a three-way within-subject double-blind active-controlled crossover design. Because of our previous experience in treating patients with ketamine, we implemented a protocol of administering 4 mg of oral ondansetron 1 h prior to dosing, to reduce the incidence of nausea and vomiting. A Data Safety and Monitoring Committee meeting was held following two early drop-outs and concerns that the 1 mg/kg ketamine dose was tolerated poorly due to distressing dissociation. This prompted a change in the randomisation sequence after five participants to ensure that 1 mg/kg ketamine was only administered following an earlier 0.5 mg/kg ketamine dose. Prior to the change in randomisation schedule, there were six dose randomisation permutations and afterwards there were three. This decision was specific to the OCD cohort and did not apply to the wider study.

There were three dosing sessions; each session was separated by at least 1 week (with the option of delaying treatment if Y-BOCS scores remained low following the previous week’s dosing). A 10-min relaxation EEG test was obtained pre-dose, and 2 and 24 h after each dosing session to assess the timing of EEG changes in response to study treatments (data to be presented elsewhere). OCD ratings and assessments of safety and tolerability were collected up to 168 h (1 week) after each dose by S.N., A.B., C.M., R.D.-B. and B.K. (research nurses) who were present during the dosing period but were blind to the treatment allocated. Patients were monitored in the research clinic for a minimum of 2 h post-dose, with vital signs obtained pre-dose and at 15-, 30-, 45-, 60-, 90-, and 120-min post-dose. OCD symptoms severity was evaluated using the Y-BOCS pre-dose, at 1-, 2-, 24- and 168-h post-dose. When used to assess response to treatment, the Y-BOCS is intended to be administered weekly but the scale developers also state that with minor modifications in wording, it can be administered at different intervals (Goodman et al., 1989). Participants were therefore asked to consider the time period since the Y-BOCS was last completed when rating their OCD. Questions 1 and 6 of the Y-BOCS ask participants to rate how many hours their obsessions and compulsions are taking/day but also offer alternative qualifiers (e.g. occasional, frequent, very frequent or near constant) that are suitable for more frequent use. Participants were directed to consider these qualifiers when completing the 1 and 2 h Y-BOCS. Responder analyses (patients with reductions in OCD scores >50%) were evaluated at 24 h post-dose because of the short-term benefits of ketamine that are typically maximal at 24 h and substantially reduced by 1 week.

Safety assessments included reported adverse events throughout the study. These could be recorded by research staff during dosing and follow-up and were categorised into serious (any event resulting in death or that is life-threatening, requires hospitalisation or results in significant disability or incapacity) and non-serious adverse events. Any serious adverse event would prompt further reporting by the principal investigator to relevant authorities. Tolerability was assessed by the Clinician-Administered Dissociative States Scale (CADSS) (Bremner et al., 1998) scores pre-dose, 30- and 60-min post-dose. Bladder symptoms were monitored using the Bladder Pain/Interstitial Cystitis Symptom Score (BPIC-SS) (Humphrey et al., 2012). Maintenance of blinding in participants and raters was not assessed. Following the randomised part of the study, participants were eligible to participate in a 6-week course of oral ketamine. Findings from this second part of the study will be reported later.

We assessed cognition using orientation questions and Trail Making tests because changes in cognition (memory impairment and executive functioning) have been reported when ketamine is used recreationally at high doses (Strous et al., 2022). Before release from the research clinic, we assessed patients’ level of orientation, blood pressure and heart rate to check vital signs were <120% of baseline, that they were able to walk unassisted, were feeling physically well and not significantly sedated or distressed. When these criteria were met, participants could be released (typically 2 h after dosing). Blinded safety data were reviewed during the study by an independent Data Safety Monitoring Board and this resulted in the change in randomisation sequence described earlier.

Summary statistics were calculated and reported for demographics, vital signs and rating scale data. Categorical variables were reported using counts and percentages. The Y-BOCS was the primary efficacy outcome measure. We calculated the sample size based on the first phase response data from Rodriguez et al. (2013). Assuming 50% of either of the ketamine treatment arms were responders at 24 h after dosing, compared with none in the placebo arm, with 12 subjects/arm and alpha = 0.05, the study has statistical power of 88%. Repeated measures analysis of variance (ANOVA) with extraction of orthogonal polynomials of dimensional factors (dose, time) was used to assess the effect of drug treatment on total Y-BOCS scores and CADSS scores. Missing Y-BOCS data (<1.5% of total) were estimated by interpolation across time based on the averages of the intact data.

## Results

**Figure 1.**
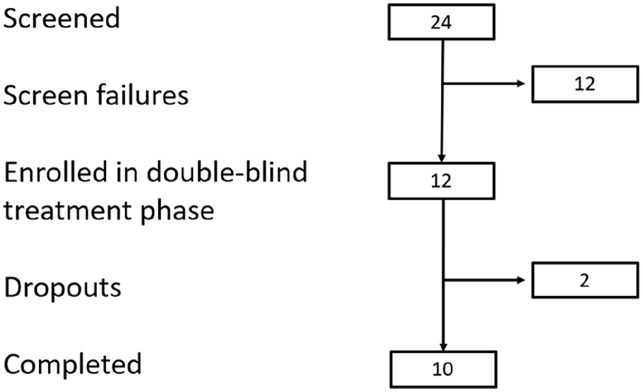
Consort diagram of participation flow.

We recruited 12 patients from a screening cohort of 24 patients (see Figure 1 for recruitment and participation details).

The main reasons for failing screening were psychiatric and physical co-morbidity consistent with the study exclusion criteria. There were two drop-outs, both of whom were unable to tolerate the acute medication side effects after one dose. Both of these participants received 1 mg/kg ketamine. Demographic and clinical characteristics of the population who received all three doses of study medication are listed in Table 1. The patients who completed the study comprised seven females and three males. Screening and treatment were undertaken between June 2021 and December 2023 and finished when the intended sample size (12 participants) had entered the study. The mean age of participants was 33 years (range 23–49 years; see Table 1), and all patients were New Zealand European. The mean (SD) baseline Y-BOCS score was 29.9 (3.9). There were high rates of psychiatric co-morbidity, primarily Major Depressive Disorder and Generalised Anxiety Disorder (see Table 1). Mean (SD) number of failed antidepressants prior to enrolment was 3.9 (1.7) (Table 1). No participants required treatment delay due to carryover effects from previous dosing.

**Table 1.** Demographic and clinical details of participants who completed the study.

Demographic	Completed participants (*n* = 10)
Mean (SD) age in years	33.4 (9.7)
Mean (SD) weight in kg	80.3 (17.8)
Gender	7 females, 3 males
Ethnicity	10 New Zealand European
Baseline (SD) Y-BOCS	29.9 (3.9)
Baseline (SD) MADRS	13.5 (4.2)
Mean (SD) failed antidepressant trials	3.9 (1.7)
Co-morbid psychiatric diagnoses^a^	Major Depressive Disorder – 7 Generalised Anxiety Disorder – 7 Social Anxiety Disorder – 4 Panic Disorder – 3 Agoraphobia – 1 Post-Traumatic Stress Disorder – 1 Alcohol Use Disorder – 1

MADRS: Montgomery Asberg Depression Rating Scale; Y-BOCS: Yale-Brown Obsessive-Compulsive Scale.

a Historical diagnoses of Major Depressive Disorder and alcohol use disorder. All other diagnoses were current.

**Figure 2.**
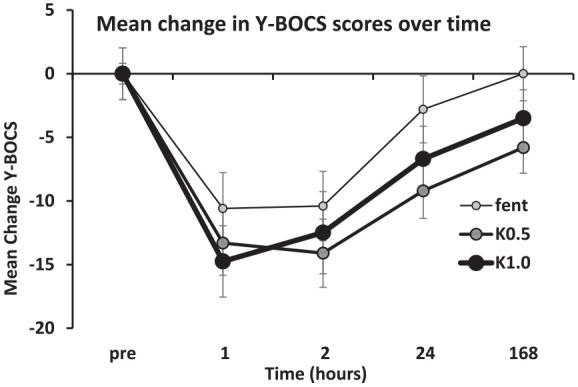
Mean change in Y-BOCS scores over time. The data shown are after subtraction of the pre-drug score to make the trends clearer but standard errors are shown from the raw data. Fent: fentanyl; K0.5: ketamine 0.5 mg/kg; K1.0: ketamine 1.0 mg/kg; Y-BOCS: Yale-Brown Obsessive-Compulsive Scale.

The mean change in Y-BOCS scores over time by study treatment for subjects who completed all three treatments is shown in Figure 2. The reductions in Y-BOCS scores were generally greater for both ketamine doses than fentanyl (dose [linear], *F*(1, 9) = 6.5, *p* = 0.031). Scores change for all treatments were maximal at 1–2 h (time [quadratic] and time [cubic], *F*(1, 9) > 30, *p* < 0.001) with a steady separation of scores over time out to 168 h that was clearest in the 0.5 mg/kg case (dose [quadratic] × time [linear], *F*(1, 9) = 6.1, *p* = 0.036) – see also response data below. Post hoc ANOVAs found a post-pre difference at 24 h between ketamine 0.5 mg/kg and fentanyl (*F*(1, 9) = 8.0, *p* = 0.020), between ketamine 1 mg/kg and fentanyl (*F*(1, 9) = 8.5, *p* = 0.017) but not between the two ketamine doses (*F*(1, 9) = 0.7, *p* = 0.443). The proportion of treatment responders (>50% reduction at 24 h compared with baseline) was 10% after fentanyl, 60% after ketamine 0.5 mg/kg and 18% after ketamine 1.0 mg/kg.

## Safety and tolerability

**Figure 3.**
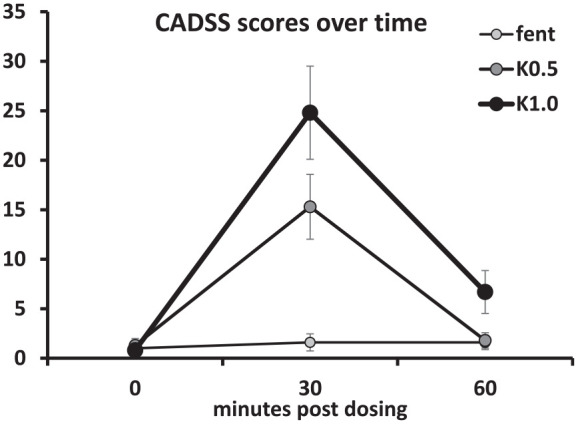
CADSS scores over time by treatment group. Bars are two standard errors. CADSS: Clinician-Administered Dissociative States Scale; Fent: fentanyl; K0.5: ketamine 0.5 mg/kg; K1.0: ketamine 1.0 mg/kg.

Thirty minutes after ketamine dosing, mean changes (SE) in systolic blood pressure were −12 (255), 1 (131) and 12 (277) mmHg for the fentanyl, ketamine 0.5 and 1.0 mg/kg dose groups, respectively, and −5 (64), 7 (69) and 8 (46) mmHg respectively for diastolic blood pressure. Blood pressure changes had essentially normalised by 60 min. All patients reported dissociative symptoms after ketamine dosing, starting approximately 3–5 min after each IM injection, with peak intensity around 15–30 min, and then slowly decreasing. CADSS scores were highest after the 1.0 mg/kg dose of ketamine (Figure 3) with both ketamine doses peaking at 30 min post-dose (drug [linear], *F*(1, 9) = 19.28, *p* = 0.002; drug [linear × time [quadratic], *F*(1, 9) = 20.45, *p* = 0.001). There were negligible dissociative effects with fentanyl.

## Adverse events

The most commonly reported adverse event after dosing with ketamine was dissociation – usually starting within 3–5 min of ketamine being administered. Many patients reported having blurred vision, experiencing a feeling of being lightheaded or sedated, and that their lips felt numb. Because of pre-dosing with ondansetron, it is possible that rates of nausea and vomiting were reduced. The most commonly experienced adverse event after fentanyl was a mild level of drowsiness that was short-lived. Mean BPIC-SS scores taken pre-dose were 2.5, 2.6 and 3.3, for the fentanyl, ketamine 0.5 and 1 mg/kg dose groups respectively. No patients experienced a serious adverse event but two patients dropped out due to not tolerating the acute dissociative effects of the 1 mg/kg dose.

## Discussion

New and effective treatments for OCD are needed because of inadequate responses to existing treatments, and the high burden of illness. We report that two doses of IM ketamine were more efficacious than IM fentanyl for OCD. Ketamine was associated with dose-dependent dissociative symptoms and cardiovascular changes, and two participants dropped out of the study after their first dose due to not tolerating ketamine at the 1 mg/kg dose.

Ketamine is a NMDA receptor antagonist with a wide range of other actions. Its principal metabolites include norketamine and hydroxy-norketamine (Highland et al., 2021). Whilst treatment-resistant depression is the most studied indication for ketamine, the range of possible therapeutic targets includes bipolar disorder, substance use disorders, eating disorders, anxiety disorders, post-traumatic stress disorder, OCD and suicidal ideation (Walsh et al., 2021). The systematic review by Walsh et al. (2021) also reported that there were multiple unanswered questions in the body of ketamine research but despite methodological limitations to existing studies, further research is warranted given the broad spectrum of potential applications and limited adverse effects.

Participants were recruited because OCD was their primary concern. The study sample was highly co-morbid which is in keeping with epidemiological evidence and suggests similarities with ‘real world’ settings. Although both ketamine doses were efficacious compared to fentanyl, the dropouts require consideration. Our clinical impression is that the acute dissociative effects of 1 mg/kg ketamine were particularly challenging for patients with OCD for whom loss of control was distressing (compared to patients with major depressive disorder and post-traumatic stress disorder) (Glue et al., 2024). Participants who received 0.5 mg ketamine prior to 1 mg/kg did not drop out from the study suggesting that familiarity with ketamine at a lower dose may enhance tolerability for higher doses. We did not experience similar dosing issues for the other diagnostic cohorts in the wider study (Beaglehole et al., 2024; Glue et al., 2024). However, we remain unclear as to the optimal ketamine dose to treat OCD. Consequently, we recommend future studies consider starting OCD patients on lower ketamine doses initially and check for response and tolerability before trialling a higher dose. We also suggest there is merit in using other strategies to improve tolerability. For example, delivering ketamine by slow IV infusion or orally to minimise dissociation.

Although the 24-h post-dose analysis did not find a significant difference in Y-BOCS scores between ketamine doses, there were more responders following 0.5 mg/kg IM ketamine than 1 mg/kg ketamine. Therefore, in addition to being more tolerable, it is possible that a dose of 0.5 mg/kg IM ketamine is more suitable than 1 mg/kg for treatment-refractory OCD. The positive benefits of ketamine on the Y-BOCS were observed at 1 h (when acute dissociation was resolving) and were also present at 2 and 24 h post-dosing. There appeared to be residual effects at 168 h post-dosing but these were not statistically significant. This confirms the relatively short-lived benefits of ketamine treatment (i.e. no more than a week for most disorders). Given the chronic burden of OCD, further work is required to establish whether ketamine has a longer-term role in OCD treatment. Areas to clarify include the optimal dose of ketamine, benefits of repeated dosing, relapse rates following courses of ketamine treatment and the longer-term safety and tolerability profile of ketamine. In our view, repeated oral dosing is likely to be a more practical solution than repeated parenteral dosing due to improved tolerability and ease of administration (Beaglehole et al., 2023).

A goal of this study was to determine if there are short-term benefits of ketamine for OCD. We believe this is an important first step prior to any longer-term studies. Consequently, parenteral as opposed to oral ketamine was required as oral ketamine is often associated with slower or delayed response (Meshkat et al., 2023). We chose to deliver ketamine by the IM route (unlike the Rodriguez et al. study that used an IV infusion) because the IM route is easy to administer and does not require IV access. Other feasible routes include sub-cutaneous (SC) injections and IV injections. An ascending dose study by Loo et al. (2016) compared IV, SC and IM routes of delivery and reported that these achieved similar antidepressant effects despite higher plasma ketamine levels for IV dosing.

Our study offered participants who completed all three study doses an optional 6-week continuation period of oral treatment (this took place after all follow-up data for the RCT had been collected). All eligible participants took advantage of this offer. The findings from this phase of the study will shed some light on the benefits of repeated oral dosing for OCD but are yet to be analysed and will be reported elsewhere.

### Limitations

In this paper, we report findings from the initial randomised crossover phase of our study. Although we report that OCD responds to ketamine in the short term in our community sample, the chronic nature of OCD suggests longer-term interventions require evaluation. In general, improved outcomes occur when psychotherapy is combined with medication interventions. We suggest that researchers consider evaluating the impact of additional psychotherapy (Kew et al., 2023) and longer ketamine courses on OCD. We did not assess blinding of participants and raters but issues with expectation bias and failure of blinding in ketamine and psychedelic trials are well-recognised (Muthukumaraswamy et al., 2021). The crossover design of this study accentuates the difficulty in maintaining blinding because each participant receives all treatments over the study period and can make comparisons between treatments. Midazolam is commonly used as a control medication in ketamine studies but this fails to adequately preserve study blinding. Consequently, fentanyl was selected at a dose commonly used for anaesthetic pre-medication in the hope that this would be a safe and suitable control. The prominent differences in dissociation scores measured by the CADSS suggest that fentanyl 50 µg also did not provide an adequate blind and we would not recommend its use for this purpose in future studies (but do not have a suitable alternative to recommend). The treatment-resistant nature of the participants provides some reassurance of the value of the ketamine-related changes we observed. However, positive expectation bias for ketamine and the blinding limitations are an alternative and additional explanation for the positive findings we report. EEG work to be published later may provide a biological explanation for ketamine responsiveness beyond that achieved through any expectation bias. In our view, longer-term studies in which all participants receive ketamine but are randomised to receive varying doses may be best suited to manage expectation effects and evaluate for dose-responsiveness.

In conclusion, our study provides further preliminary evidence for the effectiveness of ketamine in the treatment of OCD. More work is required to clarify the dosing regimen that optimises tolerability and efficacy. Additionally, longer-term studies are also required to clarify the ongoing role of ketamine treatment for OCD but our findings are encouraging given the well-known limitations that exist for treatments in this area.

## Supplemental Material

sj-docx-1-jop-10.1177_02698811241301215 – Supplemental material for Ketamine for treatment-resistant obsessive-compulsive disorder: Double-blind active-controlled crossover study Supplemental material, sj-docx-1-jop-10.1177_02698811241301215 for Ketamine for treatment-resistant obsessive-compulsive disorder: Double-blind active-controlled crossover study by Ben Beaglehole, Paul Glue, Shona Neehoff, Shabah Shadli, Neil McNaughton, Bridget Kimber, Chrissie Muirhead, Aroha de Bie, Rachel Day-Brown and Natalie J Hughes-Medlicott in Journal of Psychopharmacology
